# Association of HbA1c with functional outcome by ischemic stroke subtypes and age

**DOI:** 10.3389/fneur.2023.1247693

**Published:** 2023-09-28

**Authors:** Jihyun Jeong, Jae Kyung Park, Young Ho Koh, Jong-Moo Park, Hee-Joon Bae, Sang-Moon Yun

**Affiliations:** ^1^Department of Biostatistics, Korea University College of Medicine, Seoul, Republic of Korea; ^2^Division of Injury Prevention and Control, Bureau of Chronic Disease Prevention and Control, Korea Disease Control and Prevention Agency, Cheongju-si, Republic of Korea; ^3^Division of Brain Diseases Research, Department of Chronic Disease Convergence Research, Korea National Institute of Health, Cheongju-si, Republic of Korea; ^4^Department of Neurology, Uijeongbu Eulji Medical Center, Eulji University School of Medicine, Uijeongbu-si, Republic of Korea; ^5^Department of Neurology, Cerebrovascular Center, Seoul National University College of Medicine, Seoul National University Bundang Hospital, Seongnam, Republic of Korea; ^6^Division of Cardiovascular Disease Research, Department of Chronic Disease Convergence Research, Korea National Institute of Health, Cheongju-si, Republic of Korea

**Keywords:** hyperglycemia, HbA1c, stroke prognosis, functional loss, ischemic stroke subtype

## Abstract

**Objectives:**

To determine whether high HbA1c levels are related to short-and long-term functional outcomes in patients with ischemic stroke (IS) and whether this association differs according to the IS subtype and the patient’s age.

**Methods:**

The data of 7,380 IS patients admitted to 16 hospitals or regional stroke centers in South-Korea, between May 2017 and December 2019, were obtained from the Clinical Research Collaboration for Stroke-Korea-National Institute of Health database and retrospectively analyzed. Among these patients, 4,598 were followed-up for one-year. The HbA1c levels were classified into three groups (<5.7, 5.7 to <6.5%, ≥6.5%). Short-and long-term poor functional outcomes were defined using the modified Rankin Scale score of 2 to 6 at three-months and one-year, respectively. IS subtypes were categorized according to the Trial of ORG 10172 in Acute Stroke Treatment (TOAST) classification.

**Results:**

There was an association between higher HbA1c (≥6.5%) and poor functional outcomes at three-months in all patients (three-months; OR, 1.299, 95% CI 1.098, 1.535, one-year; OR, 1.181, 95% CI 0.952, 1.465). When grouped by age, the associations after both 3 months and 1 year observed in younger adult group (<65 years), but not in group aged 65 years and older (three-months; <65 years OR, 1.467, 95% CI 1.112, 1.936, ≥65 years OR, 1.220, 95% CI 0.987, 1.507, p for interaction = 0.038, one-year; <65 years OR, 1.622, 95% CI 1.101, 2.388, ≥65 years OR, 1.010, 95% CI 0.778, 1.312, p for interaction = 0.018). Among younger adult group, the higher HbA1c level was related to short-and long-term functional loss in patients with the small vessel occlusion subtype (three-months; OR, 2.337, 95%CI 1.334, 4.095, one-year; OR, 3.004, 95% CI 1.301, 6.938). However, in patients with other TOAST subtypes, a high HbA1c level did not increase the risk of poor outcomes, regardless of the age of onset.

**Conclusion:**

High HbA1c levels increase the risk of short-and long-term poor functional outcomes after IS onset. However, this association differs according to stroke subtype and age. Thus, pre-stroke hyperglycemia, reflected by HbA1c, may be a significant predictor for a poor prognosis after ischemic stroke, particular in young- and middle-aged adults.

## Introduction

Stroke remains a serious global health problem (1–3) and is estimated to affect more than 101 million people worldwide (4). Although stroke mortality has decreased over the past two decades (5), the number of patients with stroke has been steadily increasing, resulting in an increased economic burden due to the rising need for stroke after-care (6). Diabetes mellitus (DM) is considered a prognostic factor as well as the risk factor for stroke. Stroke outcomes are exacerbated by DM, which increases mortality and disability (7, 8). HbA1c, a glucose indicators, is used as a reliable marker to diagnose DM and assess the effects of chronic hyperglycemia in patients with stroke (9). Although several studies (10–17) have used HbA1c markers to assess the association between high glucose levels and functional loss, few have investigated the effects of HbA1c levels on poor long-term functional outcomes.

According to stroke subtypes, denoted by the Trial of ORG10172 in Acute Stroke Treatment (TOAST) classification, the profiles for stroke risk (18), prognosis (19), and functional loss (20) differ. DM and hyperglycemia are independent risk factors for stroke (21), but not for all ischemic stroke (IS) subtypes. Previous studies have suggested that DM is a risk factor in Large Artery Atherosclerosis (LAA) and Small Vessel Occlusion (SVO), but not cardioembolism (CE) (22, 23). Moreover, since IS is a heterogeneous disease (12, 24), DM (25) and hyperglycemia (26–28) may have different associations with poor outcomes by stroke subtype. Aging is not only a risk factor for stroke (29) but also a prognostic factor for functional outcomes (30). However, it is difficult to distinguish between the direct effects of age and age-associated effects on functional loss such as ischemic heart disease, hypertension, DM, and altered cognitive capacity (30–33). There is a need to assess the association between hyperglycemia-poor outcomes and age because DM is an age-related disease (34).

In the present study, we investigated the association between HbA1c levels and short-term (three-months) and long-term (one-year) functional outcomes in IS patients. We also determined whether this association differs by IS subtype and age (<65 vs. ≥65 years).

## Methods

### Study design and data collection

The study population was derived from the Clinical Research Collaboration for Stroke in Korea (CRCS-K)-National Institute of Health (NIH) database. The CRCS-K-NIH is a prospective, multicenter, web-based cohort study for ischemia stroke patients supported by a grant of the Korea Disease Control and prevention Agency. The data from patients diagnosed with acute stroke and transient ischemic attack (TIA) who were admitted to 16 hospitals or regional stroke centers in South-Korea, between May 2017 and December 2019, were obtained and retrospectively analyzed. The institutional review boards approved this study. Written, informed consent was obtained from patients and caregivers. The participants also recorded mRS score and CVD events at three-months and one-year after the stroke index (Supplementary Figure S1). The CRCS-K-NIH is a follow-up study from the previous CRCS-K study that was a prospective, nationwide, multicenter, web-based acute stroke registry of consecutive patients with acute IS admitted to 16 hospitals in South-Korea since April 2008 (35, 36). The CRCS-K study forms part of the multinational Assessment of Real-World Evidence in Stroke/TIA program and complements the SOCRATES (Acute Stroke or Transient Ischemic Attack Treated with Aspirin or Ticagrelor and Patient Outcomes) trial (37). Information on baseline demographics, medical history, risk factors, laboratory findings, stroke characteristics, prescribed medications, and post-stroke cardiovascular events was obtained from the CRCS-K database (38). CVD events during hospitalization and up to one-year after the index stroke were captured by medical record review and telephone interviews by experienced stroke coordinators in each center (38, 39).

### Study participants

**Figure 1:**
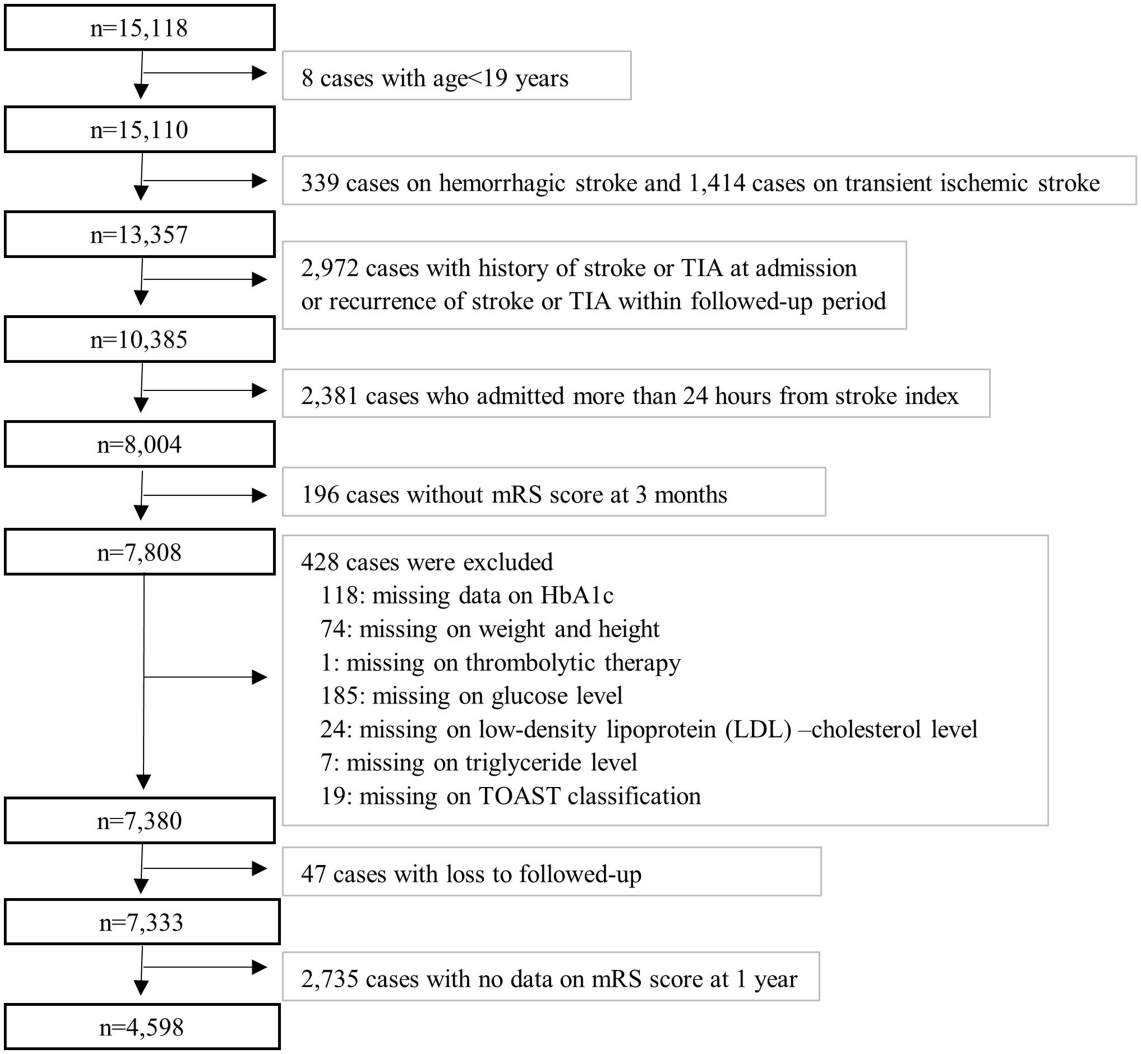
A flow chart representing the study population.

Among the 15,118 patients registered in the CRCS-K-NIH study, between May 2017 and December 2019, we identified the final IS patients following the criteria. Patients with transient symptoms with positive MRI were classified as stroke patients. Patients with posterior circulation presented symptoms of headache, dizziness, nausea, or trunk ataxia was included. We excluded the following patients: aged <19 years (*n* = 8), patients with hemorrhagic stroke or TIA (*n* = 1,753), patients with history of stroke (*n* = 2,709) or TIA (*n* = 143) at admission, and recurrence of stroke (*n* = 107) or TIA (*n* = 13) within followed-up period, hospitalization for more than 24 h from onset (*n* = 2,381), missing data on modified Rankin Scale (mRS) score at three-months (*n* = 94), and follow-up loss at three-months (*n* = 102). We also excluded missing data on other covariates (*n* = 428). Finally, we used data from 7,380 patients to assess an association between HbA1c levels and poor outcomes at three-months (short-term). When we assessed HbA1c effect for long-term (one-year) outcomes, we additionally excluded follow-up loss at one-year (*n* = 47) and no data on the mRS score at one-year (*n* = 2,735). Finally, the data of 4,598 patients were used to assess the association between hyperglycemia and long-term functional outcomes among IS patients, not include TIA and hemorrhagic stroke (Figure 1). With an approval from the Ethics Committee, clinical data were obtained from the selected participants from the CRCS-K-NIH study.

### Clinical assessment and study outcome

Hypertension, dyslipidemia, and atrial fibrillation (AF) were diagnosed on the basis of the history of each disease. DM was defined as; (1) a history of DM, (2) take antidiabetic drugs. Smoking was defined as “Yes” or “No” and those who answered, “Smoking status = Yes” also responded about “current smoker,” “Smoking cessation more than 5 years” or “Smoking cessation within 5 years.” The HbA1c levels were categorized by the criteria of the American Diabetes Association (40) and the Korean Diabetes Association (41) as follows: low (HbA1c < 5.7%), middle (5.7% ≤ HbA1c < 6.5%) and high (6.5% ≤ HbA1c). We classified IS into LAA, SVO, CE, other determined etiologies, and undetermined etiologies using the Trial of Org 10,172 in Acute Stroke Treatment (TOAST) criteria (42) in the present study. The mRS, modified Rankin disability scale is a clinician reported measure of global disability (43). The mRS score was measured face-to-face and telephone surveys by trained operator (44, 45). The mean data collection windows were 3 months±6 days, 1 year ±15 days. The mRS was used to evaluate short-and long-term functional outcomes at three-months and one-year, respectively. Considering the ability to perform outdoor activities, a poor outcome was defined as an mRS score of 2–6, and a favorable outcome as an mRS score of 0 or 1 (46, 47).

### Statistical analysis

The clinical and demographic differences at baseline were showed by HbA1c group using the t-test and $\chi^{2}$ test. Multivariate-adjusted ORs and 95% CIs for poor outcomes (mRS score = 2–6) were analyzed by logistic regression analysis. We adjusted for the following variables for poor outcome at three-months: age, sex, National Institutes of Health Stroke Scale (NIHSS) score, IS subtype (TOAST classification), systolic blood pressure (SBP), dyslipidemia, AF, body mass index (BMI), glucose level, hypertension, low-density lipoprotein (LDL) -cholesterol, and triglycerides.

We added stroke recurrence at three-months to assess HbA1c effect for one-year poor outcomes. Subgroup analysis for age (<65 or ≥ 65 years) and TOAST classification (LAA, SVO, CE, other determined and undetermined) was conducted. Statistical significance was set at *p*-value <0.05 SAS software (version 9.4; SAS Institute Inc., Cary, NC, United States) was used for all statistical analyses.

## Results

### Baseline characteristics of participants

The baseline characteristics of the total patients are presented in Supplementary Table S1. The mean age was 68.02 years, and the proportion of male patients (59.02%) was higher than the proportion of female patients (40.98%). Among patients with IS stroke, LAA was the most common subtype (31.73%), followed by undetermined (22.76%), CE (21.91%) and SVO (19.86%).

Table 1 shows the clinical and demographic characteristics of the patients according to their HbA1c levels. Among the 7,380 patients, 1,840 (24.93%) had high HbA1c at baseline. The mean age was highest in the middle HbA1c level group (low; 66.57, mid; 69.85, high; 67.75 years, *value of p* <0.0001). SBP, triglyceride levels and BMI were higher in patients with high HbA1c levels. The history of hypertension and dyslipidemia was high in the patients with high HbA1c levels. The proportion of patients with poor functional outcomes (mRS score = 2–6) was higher in the high HbA1c group at three-months and one-year, respectively (Supplementary Figure S2).

**Table 1:** Clinical characteristics according to HbA1c levels at baseline.

	HbA1c			
	Low (<5.7%)	Mid (5.7 to <6.5%)	High (≥6.5%)	*p*-value
Variables	(*n* = 2,947)	(*n* = 2,593)	(*n* = 1,840)	
Age (year), mean ± sd	66.57 ± 15.12	69.85 ± 12.08	67.75 ± 11.86	<0.0001
SBP (mmHg), mean ± sd	149.25 ± 28.04	149.40 ± 27.89	152.53 ± 29.16	<0.0001
DBP (mmHg), mean ± sd	85.51 ± 16.80	84.88 ± 16.38	85.08 ± 16.79	0.0125
LDL (mg/dL), mean ± sd	107.38 ± 33.36	109.54 ± 35.89	109.76 ± 40.37	<0.0001
TG (mg/dL), mean ± sd	112.83 ± 83.40	125.06 ± 73.29	152.24 ± 109.21	<0.0001
Initial random glucose level at admission (mg/dL)	119.53 ± 27.49	130.31 ± 35.28	205.32 ± 85.52	<0.0001
BMI (kg/m^2^), mean ± sd	23.27 ± 3.49	24.01 ± 3.62	24.49 ± 3.65	<0.0001
Sex, *n* (%)				
Male	1743 (59.14)	1,483 (57.19)	1,130 (61.41)	0.0187
Female	1,204 (40.86)	1,110 (42.81)	710 (38.59)	
TOAST classification, *n* (%)				
LAA	809 (27.45)	801 (30.89)	732 (39.78)	<0.0001
SVO	618 (20.97)	489 (18.86)	359 (19.51)	
CE	659 (22.36)	634 (24.45)	324 (17.61)	
Other determined	159 (5.40)	79 (3.05)	37 (2.01)	
Undetermined	702 (23.82)	590 (22.75)	388 (21.09)	
Hypertension, *n* (%)				
No	1,264 (42.89)	886 (34.17)	528 (28.70)	<0.0001
Yes	1,683 (57.11)	1707 (65.83)	1,312 (71.30)	
Diabetes*, *n* (%)				
No	2,792 (94.74)	2,152 (82.99)	253 (13.75)	<0.0001
Yes	155 (5.26)	441 (17.01)	1,587 (86.25)	
Dyslipidemia, *n* (%)				
No	2,367 (80.32)	1870 (72.12)	1,190 (64.67)	<0.0001
Yes	580 (19.68)	723 (27.88)	650 (35.33)	
History of smoking, *n* (%)				
No	1858 (63.05)	1,669 (64.37)	1,124 (61.09)	0.0835
Yes	1,089 (36.95)	924 (35.63)	716 (38.91)	
Smoking status, *n* (%)				
Current smoker	731 (67.13)	618 (66.88)	484 (67.60)	0.9610
Smoking cessation ≥5 years	262 (24.06)	227 (24.57)	176 (24.58)	
Smoking cessation <5 years	96 (8.82)	79 (8.55)	56 (7.82)	
Atrial fibrillation, *n* (%)				
No	2,331 (79.10)	1945 (75.01)	1,515 (82.34)	<0.0001
Yes	616 (20.90)	648 (24.99)	325 (17.66)	
Acute Thrombolytic, *n* (%)				
No	2,218 (75.26)	1995 (76.94)	1,472 (80.00)	0.0076
IV	344 (11.67)	259 (9.99)	170 (9.24)	
IA	204 (6.92)	181 (6.98)	97 (5.27)	
IV + IA	181 (6.14)	158 (6.09)	101 (5.49)	
NIHSS, *n* (%)				
0	774 (26.26)	713 (27.5)	451 (24.51)	0.0024
1,2	617 (20.94)	596 (22.98)	434 (23.59)	
3,4,5,6	729 (24.74)	583 (22.48)	494 (26.85)	
≥7	827 (28.06)	701 (27.03)	461 (25.05)	

sd, standard deviation; SBP, systolic blood pressure; DBP, diastolic blood pressure; TG, triglyceride; BMI, body mass index; TOAST, Trial of ORG 10172 in Acute Stroke Treatment; LAA, Large Artery Atherosclerosis; SVO, Small Vessel Occlusion; CE, Cardioembolism; Other determined: Other determined Etiology; Undetermined: Undetermined Etiology; IV: intravenous; IA: intra-arterial; NIHSS: National Institute of Health stroke scale. *Diabetes was defined by; (1) the history of DM, (2) take antidiabetic drug.

### Elevated HbA1c associated with poor functional outcomes

In Table 2, a high HbA1c level related to short-term poor outcomes among entire IS patients, but not to long-term poor outcomes (three-months: OR 1.299, 95% CI 1.098, 1.535, one-year: OR 1.181, 95% CI 0.952, 1.465). We investigated the association between HbA1c-poor outcomes by age groups to assess to age associated effect. In Table 3, the risk of poor outcome was significantly increase in <65 aged group (three-months: OR 1.467, 95% CI 1.112, 1.936, one-year: OR 1.622, 95% CI 1.101, 2.388). However there was no significant association in the ≥65 aged group. There was an interaction effect between age and HbA1c level for the short and long term poor functional outcomes (three-months: *p* = 0.038, one-year: *p* = 0.018, Table 3).

**Table 2:** Odds Ratios (95% CIs) for poor functional outcomes according to HbA1c levels.

HbA1c^*^	3 months outcome		1-year outcome	
	Favorable / Poor	Multivariate Adjusted (Model1)^**^	Favorable / Poor	Multivariate Adjusted (Model2)^***^
Low	1537/1410	1 (ref)	996/826	1(ref)
Mid	1282/1311	1.141 (1.006, 1.294)	832/792	1.158 (0.984, 1.364)
High	842/998	1.299 (1.098, 1.535)	581/571	1.181 (0.952, 1.465)

^*^HbA1c level; Low: <5.7%, Mid: 5.7 to <6.5%, High: ≥6.5%. ^**^Model1 is on 3-months poor outcome adjusted for age, sex, NIHSS, IS subtype (TOAST classification), SBP, dyslipidemia, AF, BMI, glucose level, hypertension, LDL-cholesterol, triglyceride. ^***^Model2 is on 1-year poor outcome adjusted for variables in model1 plus 3-months stroke recurrence.

**Table 3:** Odds Ratios (95% CIs) for poor functional outcomes according to HbA1c levels, stratified by age group.

	HbA1c^*^	3 months outcome			1-year outcome		
		Favorable/ Poor	Multivariate Adjusted (Model1)^**^	P for interaction	Favorable/ Poor	Multivariate Adjusted (Model2)^***^	P for interaction
<65 years	Low	845/379	1 (ref)	0.038	543/180	1(ref)	0.018
	Mid	540/281	1.234 (0.995, 1.531)		345/138	1.305 (0.960, 1.775)	
	High	416/292	1.467 (1.112, 1.936)		287/139	1.622 (1.101, 2.388)	
≥65 years	Low	692/1031	1 (ref)		453/646	1(ref)	
	Mid	742/1030	1.083 (0.926, 1.267)		487/654	1.070 (0.880, 1.302)	
	High	426/706	1.220 (0.987, 1.507)		294/432	1.010 (0.778, 1.312)	

^*^Hba1c level; Low: <5.7%, Mid: 5.7% to < 6.5%, High: ≥6.5%. ^**^Model1 is on 3-months poor outcome adjusted for age, sex, NIHSS, IS subtype (TOAST classification), SBP, dyslipidemia, AF, BMI, glucose level, hypertension, LDL-cholesterol, triglyceride. ^***^Model2 is on 1-year poor outcome adjusted for variables in model1 plus 3-months stroke recurrence.

### Elevated HbA1c associated with poor functional outcomes by stroke subtypes and age

We also assessed the association between HbA1c-poor outcomes by TOAST classification and age. In all age patients, the association was noted for short-and long-term in SVO subtype (SVO, three-months: OR 1.652, 95% CI 1.138, 2.397, one-year: OR 1.814, 95% CI 1.091, 3.015; Supplementary Table S2). In patients with other TOAST subtypes, a high HbA1c level did not increase the risk of poor outcomes. In patients aged <65 years, HbA1c had an increasing risk of short-and long-term poor outcomes in SVO (SVO, three-months: OR 2.337, 95% CI 1.334, 4.095, one-year: OR 3.004, 95%CI 1.301, 6.938; Table 4). However, there was no significant effect in the older adult patients with SVO (Supplementary Table S3).

**Table 4:** Odds Ratios (95% CIs) for functional outcomes according to HbA1c levels by TOAST classification among young patients.

IS subtype (<65 years)	HbA1c^*^	3 months outcome		1-year outcome	
		Favorable/Poor	Multivariate Adjusted (Model1)^**^	Favorable/Poor	Multivariate Adjusted (Model2)^***^
-LAA	Low	210/113	1 (ref)	136/48	1(ref)
	Mid	179/91	0.940 (0.644, 1.373)	116/50	1.287 (0.757, 2.187)
	High	171/114	1.231 (0.777, 1.952)	122/58	1.554 (0.818, 2.953)
-SVO	Low	245/58	1 (ref)	154/20	1(ref)
	Mid	152/52	1.610 (1.015, 2.551)	96/14	1.394 (0.643, 3.023)
	High	125/71	2.337 (1.334, 4.095)	85/27	3.004 (1.301, 6.938)
-CE	Low	121/53	1 (ref)	70/31	1(ref)
	Mid	64/44	1.743 (0.910, 3.342)	39/24	1.235 (0.491, 3.102)
	High	32/30	1.690 (0.663, 4.310)	22/13	1.225 (0.321, 4.678)
-Other determined	Low	78/48	1 (ref)	56/25	1(ref)
	Mid	28/21	1.039 (0.441, 2.446)	22/12	1.811 (0.590, 5.562)
	High	8/12	1.745 (0.460, 6.619)	6/10	3.637 (0.517, 25.600)
-Undetermined	Low	191/107	1 (ref)	127/56	1(ref)
	Mid	117/73	1.140 (0.736, 1.765)	72/38	1.081 (0.587, 1.991)
	High	80/65	1.171 (0.647, 2.121)	52/31	1.203 (0.530, 2.729)

TIA, transient ischemic attack; LAA, Large Artery Atherosclerosis; SVO, Small Vessel Occlusion; CE, Cardioembolism; Other determined: Other determined Etiology; Undetermined: Undetermined Etiology. ^*^HbA1c level; Low: <5.7%, Mid: 5.7 to < 6.5%, High: ≥6.5%. ^**^Model1 is on 3-months poor outcome adjusted for age, sex, NIHSS, SBP, dyslipidemia, AF, BMI, glucose level, hypertension, LDL-cholesterol, triglyceride. ^***^Model2 is on 1-year poor outcome adjusted for variables in model1 plus 3-months stroke recurrence.

## Discussion

To the best of our knowledge this study is the first to investigate the association between high HbA1c levels with short-and long-term functional outcomes according to the TOAST classification and age. There was the association between higher HbA1c (≥6.5%) and poor functional outcome at three-months and one-year among younger adult patients (<65 years). In the subgroup analysis, higher HbA1c was associated with short-and long-term functional loss in the SVO subtype among the <65 age group.

We showed that high admission HbA1c level (≥6.5%) is associated with poor functional outcomes after three-months in all patients with IS. When grouped by age, the associations after both 3 months and 1 year were particularly evident in patients with IS who were younger than 65 years but not in patients who were older than 65 years. Interactions between hyperglycemia and age were observed for short-and long functional outcomes. The association between hyperglycemia and functional outcomes may be affected by patient age, which is explained by the potential ceiling effect on the functional outcomes of chronic hyperglycemia in older adults (48). Because older age is a critical predictor of poor outcome factors in ischemic and hemorrhagic stroke (49, 50), the association between HbA1c levels and poor outcomes is not sufficiently challenging for older age. Therefore, a worsening prognosis due to elevated HbA1c levels after stroke may not be observed in older adults with high HbA1c levels. Our findings are consistent with previous results on the interaction between age and DM and functional outcomes (48, 51).

Stratified by the TOAST classification, the association between elevated HbA1c levels and short-and long-term functional outcomes was observed only in patients with SVO. Although some studies reposted no association between stress hyperglycemia, defined as fasting glucose levels at admission, and poor functional outcomes in the SVO subtype (52, 53), other studies analyzing chronic hyperglycemia, define as HbA1c level at admission, were consistent with our results (12, 54). A Chinese hospital-based study (12) showed an HbA1c-functional outcome association among patients with small artery occlusion and those with a mean age of 61.7 years. This study reported that higher HbA1c levels at admission increased poor functional loss at three-months after stroke onset, which is consistent with our data. Moreover, in the present study, when grouped by age, an association was observed in those aged <65 years but not in those aged ≥65 years.

The mechanism of an association between hyperglycemia at admission and poor functional outcome after stroke onset is still unclear (10, 12). Hyperglycemia can aggravate ischemic damage by disrupting recanalization and increasing reperfusion injury, which may be associated with poor functional outcome (55). In particular, diabetes and elevated HbA1c levels are associated with the number of lacunar infarcts, also known as SVO, less than 7 mm in diameter (56). Elevated HbA1c levels can increase blood viscosity (57, 58), and elevated blood viscosity could impair microvascular tissue perfusion (59), which may be associated with poor functional outcomes in patients with SVO.

Our study has the following strengths. First, we used data from a large sized multicenter cohort study. The large-sample size provided sufficient statistical power to examine the association between hyperglycemia and functional outcomes according to stroke subtype and age. Second, the participants were followed-up for a one-year period and we used the mRS score at one-year to assess long-term poor outcomes. To the best of our knowledge this is the first study to assess the association between high HbA1c levels and the risk of poor long-term functional outcomes in Korea.

However, despite our strengths our study has several limitations. First, we did not consider management of glucose levels using antidiabetic drugs or other treatments during hospitalization. However, we adjusted for the glucose levels at admission when an HbA1c-poor outcome association was found. Second, there may have been selection bias because we excluded data on missing HbA1c levels and other covariate variables at admission. In addition, a high number of one-year follow-up losses occurred due to patients simply not reaching one-year of follow-up owing to study closing. Third, we adjusted for confounders in our study but several confounding factors such as alcohol consumption and physical activity were not considered because this information was not included in our data. Fourth, our study includes only Korean ethnic population and results may not be generalizable to other ethnic groups. Fifth, choosing a different reasonable mRS cut-off point of favorable and poor functional outcome could have produced a different result.

## Conclusion

In this study, the high HbA1c level, especially HbA1c ≥6.5%, increased the risk of short-and long- term poor functional outcomes after stroke onset. This association was observed in patients with the SVO subtype in aged <65 years. Thus, elevated HbA1c may be a significant predictor for a poor prognosis after ischemic stroke, particular in young- and middle-aged adults.

## Data availability statement

The data analyzed in this study is subject to the following licenses/restrictions: The clinical data are available upon request following the submission of a legitimate academic research proposal to be assessed by the Clinical Research Collaboration for Stroke in Korea (CRCS-K) steering committee. Requests to access these datasets should be directed to J-MP, jmpark@eulji.ac.kr.

## Ethics statement

The studies involving humans were approved by the institutional review boards in Korea Disease Control and Prevention Agency (KDCA). The studies were conducted in accordance with the local legislation and institutional requirements. The participants provided their written informed consent to participate in this study.

## Author contributions

JJ and S-MY contributed to the conception and design of the study. J-MP and H-JB collected clinical data. JJ carried out data curation, formal analysis, and drafting of the manuscript. JP, YK, and S-MY reviewed and edited the manuscript. All authors contributed to the article and approved the submitted version.

## Funding

This research was supported by the “National Institute of Health” research project (project No. 2020-NI-025-00 and 2022-NG-007-00).

## Conflict of interest

The authors declare that the research was conducted in the absence of any commercial or financial relationships that could be construed as a potential conflict of interest.

## Publisher’s note

All claims expressed in this article are solely those of the authors and do not necessarily represent those of their affiliated organizations, or those of the publisher, the editors and the reviewers. Any product that may be evaluated in this article, or claim that may be made by its manufacturer, is not guaranteed or endorsed by the publisher.
