# Supplementary material for: Association of HbA1c with functional outcome by ischemic stroke subtypes and age
Source: Front Neurol. 2023 Sep 28;14:1247693. doi: 10.3389/fneur.2023.1247693 (PMC10568315; doi:10.3389/fneur.2023.1247693)
Supplement: Supplementary file 1 [file Data_Sheet_1.docx]

Supplementary Material

Association of HbA1c with functional outcome by Ischemic stroke subtypes and age

**Jihyun Jeong ^1, 2^, Jae Kyung Park ^3^, Young Ho Koh ^3^, Jong-Moo Park ^4^, Hee-Joon Bae ^5^, Sang-Moon Yun ^6^*****

**** Correspondence:* Sang-Moon Yun:** [ysm0304.0304@gmail.com](mailto:ysm0304.0304@gmail.com)

Supplementary Table

Supplementary Table 1. The study population’s baseline characteristics

Supplementary Table 2. HbA1C-functional outcomes according to HbA1c level by TOAST classification among patients with ischemic stroke

Supplementary Table 3. HbA1C-functional outcomes according to HbA1c levels by TOAST classification among older ischemic stroke

Supplementary Figure legend

Supplementary Figure 1. CRCS-K-NIH timeline

Supplementary Figure 2. The proportion of mRS score stratified HbAc1 level

**Supplementary Figure 1. CRCS-K-NIH Timeline**

Outcome capture at 3 months

Outcome capture at 1 year

Ongoing study

Baseline

Stroke Index

mRS score

clinical event (stroke recurrence, CVD event, other death)

2008

2017

2021

CRCS-K

CRCS-K-NIH

We used data in the present study

2017.5.~2019.12.

CRCS-K & CRCS-K-NIH

**Supplementary Figure 2. The proportion of mRS score stratified HbAc1 level**

1. mRS score at admission
2. mRS score at 3 months
3. mRS score at 1-year

**Supplementary Table 1. The study population’s baseline characteristics**

|  | Total participants (n=7380) |
| --- | --- |
| Characteristics |  |
| Age (years), mean ± sd | 68.02 ± 13.40 |
| SBP (mmHg), mean ± sd | 150.12 ± 28.30 |
| DBP (mmHg) , mean ± sd | 85.18 ± 16.65 |
| LDL (mg/dL) , mean ± sd | 108.73 ± 36.11 |
| TG (mg/dL), mean ± sd | 126.95 ± 88.75 |
| Initial random glucose level at admission (mg/dL) , mean ± sd | 144.70 ± 61.67 |
| BMI (kg/m^2^), mean ± sd | 23.83 ± 3.61 |
| Male, n (%) | 4356 (59.02) |
| TOAST classification, n (%) |  |
| -LAA | 2342 (31.73) |
| -SVO | 1466 (19.86) |
| -CE | 1617 (21.91) |
| -Other determined | 275 (3.73) |
| -Undetermined | 1680 (22.76) |
| Hypertension, n (%) | 4702 (63.71) |
| Diabetes*, n (%) | 2183 (29.58) |
| Dyslipidemia, n (%) | 1953 (26.46) |
| History of smoking, n (%) | 2729 (36.98) |
| Atrial fibrillation, n (%) | 1589 (21.53) |
| Acute Thrombolytic, n (%) |  |
| No | 5685 (77.03) |
| IV | 773 (10.47) |
| IA | 482 (6.53) |
| IV+IA | 440 (5.96) |
| NIHSS, n (%) |  |
| 0 | 1938 (26.26) |
| 1,2 | 1647 (22.32) |
| 3,4,5,6 | 1806 (24.47) |
| ≥7 | 1989 (26.95) |

sd: standard deviation; SBP: systolic blood pressure; DBP: diastolic blood pressure; TG: triglyceride; BMI: body mass index; TOAST: Trial of ORG 10172 in Acute Stroke Treatment; LAA: Large Artery Atherosclerosis; SVO: Small Vessel Occlusion; CE: Cardioembolism; Other determined: Other determined Etiology; Undetermined: Undetermined Etiology; IV: intravenous; IA: intra-arterial; NIHSS: National Institute of Health stroke scale.

*Diabetes was defined by; 1) the history of DM, 2) take antidiabetic drug.

**Supplementary Table 2. HbA1C-functional outcomes according to HbA1c levels by TOAST classification among patients with ischemic stroke.**

| IS subtype  (all age patients) | HbA1c* | 3 months outcome | | 1-year outcome | |
| --- | --- | --- | --- | --- | --- |
|  |  | Favorable/Poor | Multivariate Adjusted  (Model1)** | Favorable/Poor | Multivariate Adjusted  (Model2)*** |
| -LAA | Low | 424/385 | 1(ref) | 269/208 | 1(ref) |
|  | Mid | 399/402 | 1.204(0.960, 1.509) | 266/251 | 1.373(1.026, 1.837) |
|  | High | 337/395 | 1.223(0.925, 1.619) | 231/219 | 1.165(0.811, 1.675) |
| -SVO | Low | 417/201 | 1(ref) | 276/103 | 1(ref) |
|  | Mid | 320/169 | 1.143(0.861, 1.517) | 210/87 | 1.152(0.784, 1.691) |
|  | High | 210/149 | 1.652(1.138, 2.397) | 147/71 | 1.814(1.091, 3.015) |
| -CE | Low | 273/386 | 1(ref) | 166/246 | 1(ref) |
|  | Mid | 240/394 | 1.275(0.965, 1.684) | 155/243 | 1.093(0.765, 1.564) |
|  | High | 108/216 | 1.386(0.921, 2.084) | 78/137 | 0.978(0.593, 1.614) |
| -Other determined | Low | 87/72 | 1(ref) | 63/44 | 1(ref) |
|  | Mid | 41/38 | 0.799(0.407, 1.568) | 27/25 | 1.418(0.578, 3.480) |
|  | High | 12/25 | 1.286(0.454, 3.643) | 9/20 | 2.169(0.532, 8.839) |
| -Undetermined | Low | 336/366 | 1(ref) | 222/225 | 1(ref) |
|  | Mid | 282/308 | 1.014(0.782, 1.316) | 174/186 | 0.961(0.685, 1.350) |
|  | High | 175/213 | 1.185(0.830, 1.691) | 116/124 | 1.026(0.654, 1.609) |

LAA: Large Artery Atherosclerosis; SVO: Small Vessel Occlusion; CE: Cardioembolism; Other determined: Other determined Etiology; Undetermined: Undetermined Etiology.

* HbA1c level; Low: <5.7%, Mid: 5.7$\leq$ to <6.5%, High: ≥6.5%

** Model1 is on 3-months poor outcome adjusted for age, sex, NIHSS, SBP, dyslipidemia, AF, BMI, glucose level, hypertension, LDL-cholesterol, triglyceride

*** Model2 is on 1-year poor outcome adjusted for variables in model1 plus 3-months stroke recurrence.

**Supplementary Table 3. HbA1C-functional outcomes according to HbA1c levels by TOAST classification among older ischemic stroke**

| IS subtype  (**≥**65 years) | HbA1c* | 3 months outcome | | 1-year outcome | |
| --- | --- | --- | --- | --- | --- |
|  |  | Favorable/ Poor | Multivariate Adjusted  (Model1)** | Favorable/ Poor | Multivariate Adjusted  (Model2)*** |
| Ischemic stroke | Low | 692/1031 | 1(ref) | 453/646 | 1(ref) |
|  | Mid | 742/1030 | 1.083(0.926, 1.267) | 487/654 | 1.070(0.880, 1.302) |
|  | High | 426/706 | 1.220(0.987, 1.507) | 294/432 | 1.010(0.778, 1.312) |
| -LAA | Low | 214/272 | 1(ref) | 133/160 | 1(ref) |
|  | Mid | 220/311 | 1.363(1.023, 1.816) | 150/201 | 1.332(0.934, 1.899) |
|  | High | 166/281 | 1.296(0.906, 1.853) | 109/161 | 1.030(0.658, 1.612) |
| -SVO | Low | 172/143 | 1(ref) | 122/83 | 1(ref) |
|  | Mid | 168/117 | 0.915(0.637, 1.313) | 114/73 | 1.037(0.659, 1.634) |
|  | High | 85/78 | 1.295(0.782, 2.143) | 62/44 | 1.369(0.711, 2.635) |
| -CE | Low | 152/333 | 1(ref) | 96/215 | 1(ref) |
|  | Mid | 176/350 | 1.139(0.832, 1.560) | 116/219 | 1.010(0.680, 1.502) |
|  | High | 76/186 | 1.252(0.791, 1.979) | 56/124 | 0.875(0.506,1.514) |
| -Other determined | Low | 9/24 | 1(ref) | 7/19 | 1(ref) |
|  | Mid | 13/17 | 0.355(0.097, 1.303) | 5/13 | 0.724(0.128, 4.105) |
|  | High | 4/13 | 0.338(0.038, 2.974) | 3/10 | 0.686(0.064, 7.352) |
| -Undetermined | Low | 145/259 | 1(ref) | 95/169 | 1(ref) |
|  | Mid | 165/235 | 0.953(0.684, 1.329) | 102/148 | 0.933(0.613, 1.422) |
|  | High | 95/148 | 1.150(0.729, 1.812) | 64/93 | 0.942(0.544, 1.632) |

LAA: Large Artery Atherosclerosis; SVO: Small Vessel Occlusion; CE: Cardioembolism; Other determined: Other determined Etiology; Undetermined: Undetermined Etiology.

* Hba1c level; Low: <5.7%, Mid: 5.7%≤ and <6.5%, High: ≥6.5%

** Model1 is on 3-months poor outcome adjusted for age, sex, NIHSS, SBP, dyslipidemia, AF, BMI, glucose level, hypertension, LDL-cholesterol, triglyceride

***Model2 is on 1-year poor outcome adjusted for variables in model1 plus 3-months stroke recurrence.
